# Dietary Patterns and Lifestyle Factors Associated with the Risk of Colorectal Cancer: A Hospital-Based Case-Control Study among Malaysians

**DOI:** 10.21315/mjms2024.31.1.18

**Published:** 2024-02-28

**Authors:** Sook Yee Lim, Vaidehi Ulaganathan, Padmini Nallamuthu, Baskaran Gunasekaran, Shamala Salvamani

**Affiliations:** 1 Faculty of Applied Sciences, UCSI University, Kuala Lumpur, Malaysia; 2 Division of Applied Biomedical Science and Biotechnology, School of Health Sciences, International Medical University, Kuala Lumpur, Malaysia

**Keywords:** case-control studies, colorectal cancer, dietary patterns, smoking, physical activity, sedentary behaviour, factor analysis

## Abstract

**Background:**

This study aimed to examine the association between dietary patterns, lifestyle factors, and colorectal cancer (CRC) risk among the Malaysian population.

**Methods:**

We recruited 100 patients and 100 controls from two selected government hospitals. Principal component analysis was used to identify dietary patterns using a 123-item semiquantitative food frequency questionnaire. Tobacco smoking and alcohol consumption questionnaires were modified from the WHO STEPS Survey questionnaire. Physical activity levels were assessed using the revised Global Physical Activity questionnaire. Associations between dietary patterns, lifestyle factors and CRC risk were assessed using logistic regression with SPSS version 24.0.

**Results:**

Three dietary patterns were derived from factor analysis: i) vegetables; ii) meat, seafood and processed food; and iii) grains and legumes. High vegetable diet intake was independently and significantly associated with an 81% decreased risk of CRC (odds ratio [OR]: 0.19; 95% confidence interval [CI]: 0.08, 0.46). Both recreational-related physical activity (OR: 2.04; 95% CI: 1.14, 3.64) and vigorous physical activity (OR: 2.06; 95% CI: 1.13, 3.74) are significantly associated with decreased risk of CRC. Increasing the number of cigarettes smoked (≥ 16 cigarettes) per day significantly increased the odds of developing CRC (OR: 2.58; 95% CI: 1.95, 6.75). The duration of alcohol consumption cessation was inversely associated with CRC risk (OR: 2.52; 95% CI: 2.30, 10.57).

**Conclusion:**

The protective effects of a fruit and vegetable diet, and a healthy lifestyle can be used to develop interventions that help reduce the risk of CRC in the Malaysian population.

## Introduction

Approximately 19.3 million new colorectal cancer (CRC) cases have been reported worldwide, with half of all cases and 58.3% of the cancer deaths expected to occur in Asia by 2020 (1). Globally, CRC is the third most common cancer affecting both men and women and ranks second in mortality after lung cancer (1). In Malaysia, CRC incidence and mortality rates are higher in men than in women. The Chinese (27.35 per 100,000 populations) have the highest reported overall age standardised incidence rate, followed by Malay (18.95 per 100,000 populations) and Indian ethnicities (17.55 per 100,000 populations) (2).

The CRC incidence has decreased by adapting a healthy dietary pattern and an active lifestyle (1, 3). Therefore, knowledge of modifiable risk factors for CRC and potential interventions targeting CRC prevention is important. Because diet plays a vital role in the initiation and progression of many cancers, the effects of food consumption must be measured in combination with the risk of CRC. Pathological onset, in combination with various food and nutrients, can be distinguished only when the complete eating pattern is considered for better preventive measures (4). Congruently, together with the many ethnic groups and dietary variabilities found in Asia, factor analysis is an appropriate method for identifying nutritional patterns that could be responsible for increasing the risk of CRC in Malaysia. Studies among CRC patients have directly correlated red or processed meat with an increased risk. Conversely, calcium supplements and adequate intake of whole grains, fruits, vegetables and dairy products are inversely related to the risk of CRC (5–12).

Several systematic reviews and meta-analyses have identified an inverse relationship between physical activity and CRC risk (13–15). Epidemiological studies have also suggested that higher levels of physical activity are associated with a lower risk of CRC (16–20). However, a sedentary lifestyle, defined as sitting or reclining posture activities that expend less than or equal to 1.5 metabolic equivalents of tasks (METs) (21), showed a positive association with CRC risk (22–24). Based on numerous studies, several international agencies have classified the evidence level as ‘convincing’ as it supports the association between physical activity and CRC cancer risk (20, 25–28). However, this evidence is primarily supported by research conducted in Western populations, and there is less information about physical activity and CRC risk in Asian nations. The available data indicate that adherence to physical activity and CRC prevention recommendations are likely poor among Malaysian adults. Additionally, detailed information such as the type or duration of physical activity may provide additional information for predicting the risk of CRC and, hence, developing effective intervention programmes.

Smoking is a well-established risk factor for CRC. The toxic chemicals in cigarette smoke directly damage the colorectal mucosa, resulting in further genetic or epigenetic alterations (32, 33). A prospective study showed that cigarette cessation for more than 10 years lowered the risk of CIMP-high CRC by 50% (34), indicating that cigarette cessation may reverse the positive association between smoking and CRC risk. To further stratify the risk of CRC according to region and population, a Singapore Chinese population-based cohort study showed a positive association between tobacco smoking and rectal cancer risk (35). Conversely, a Japanese cohort study showed that pack-years of tobacco smoking increased the risk of rectal cancer in men only (36). A Korean National Health study indicated that former smokers had a higher risk of distal cancer in men but not of CRC (37). However, two studies conducted in Thailand and Oman found no significant association between cigarette smoking and the risk of CRC (38, 39).

Although frequent alcohol consumption is much less prevalent in Malaysia than in Western countries, alcohol consumption remains an important confounding factor for CRC risk. Higher alcohol consumption was associated with an increased risk of CRC compared with non-drinkers (31, 40). Acetaldehyde is the primary metabolite of ethanol and is produced by alcohol dehydrogenase. It is a carcinogen that can damage the intestinal mucosa and stimulate cell proliferation (41, 42). High alcohol consumption can result in acetaldehyde accumulation in the body (43). A Japanese study reported that 25% of male CRC cases were attributable to an alcohol intake of ≥ 23 g/day (44). A Chinese study showed that alcohol consumption increases CRC incidence and mortality rates by 8.7% in men and 1.1% in women (12). However, this relationship was not observed in two studies conducted in Thailand and Oman (38, 39). In summary, the results of studies on smoking and alcohol consumption in relation to CRC in Asian populations are inconsistent. The widely acknowledged approach in nutritional epidemiology has mainly focused on the effect of single nutrients or foods on the onset of CVD (45). However, it is well-known that nutrients and foods are consumed in combination (46). To demonstrate the effect of food consumption as a combination, several studies have used dietary pattern analysis to investigate the association with CRC risk (3, 6, 8, 10, 11, 47–49); however, the results are inconsistent, especially across different regions, cultures and backgrounds. Therefore, studying various dietary patterns in other populations and exploring their association with CRC risk is of great scientific interest. Soon and Tee (50), and Lim et al. (51, 52) reported that dietary patterns among Malaysians have shifted from traditional dietary patterns, which are high in fresh fruits and vegetables, to Western dietary patterns, that are high in processed meat, wheat, sugars, fats and salts. To date, there is limited published evidence on the association between dietary patterns and CRC risk in the Malaysian population. Therefore, this study aimed to examine the association between dietary patterns, lifestyle factors and CRC risk using a case-control study design in Malaysia.

## Methods

### Study Population

This case-control study was conducted in two public hospitals, Selayang Hospital and Hospital Kuala Lumpur, which are the leading Malaysian government hospitals that diagnose and treat most CRC cases of CRC in Malaysia. The sample size was calculated using a Power and Sample Size calculator (53). The dichotomous tab was chosen for the calculation in accordance with the study design, which used matched cases and controls with dichotomous outcomes, and alternative hypotheses were specified in terms of odds ratios (ORs). The OR was 2.61, and the correlation coefficient I for exposure between matched cases and controls was 0.2 (54). With a significance criterion of α = 0.05 and power = 0.8, the minimum sample size needed for the study was 88 patients, with one matched control per case. Thus, a sample size of 100 CRC patients in the case group and 100 cancer-free patients in the control group were adequate to test the study hypothesis. Purposive sampling was performed based on the inclusion and exclusion criteria. Patients recruited as the ‘cases’ for this study were newly diagnosed with CRC (within 6 months of diagnosis) and confirmed through colonoscopy screening examination. However, the ‘control’ participants were patients found negative for CRC and other cancers after undergoing a complete colonoscopy screening examination. To control for confounding factors, all the case patients were matched with control patients by five years of age, sex and ethnicity. Patients with a history of cardiovascular disease, renal failure, inflammatory bowel disease, other malignancies or metastases, pregnancy, mental instability, inability to communicate and those involved in other studies were excluded. Before recruitment, all study participants were given an information form and signed a consent form indicating that they understood the implications and potential risks of the study and agreed to the proposed action.

### Study Instruments

#### Interviewer Administrated Questionnaire

##### Sociodemographic background and medical history

Information on sociodemographic background and medical history was obtained through personal interviews using a pre-tested structured questionnaire. In addition to the verbal information obtained from the questionnaire session, the participants’ medical histories were extracted from each participating hospital’s records. Histopathology reports were referred to for cancer characteristics such as the site, stage, and cancer indications. Medical records also revealed the participants’ individual and family histories of any type of chronic disease, including cancer, inflammatory bowel disease, cardiovascular disease, renal failure, diabetes mellitus, hypertension and dyslipidaemia.

##### Lifestyle assessments

The tobacco smoking and alcohol consumption questionnaires were modified from the WHO STEPS Survey questionnaire (55). Participants were asked about their current smoking practices, the number of cigarettes smoked daily, and the duration of smoking. For the ex-smokers, the time of how long ago they quit smoking and the number of cigarettes they smoked in the past were inquired. Regarding alcohol consumption attitudes among participants, questions were asked about current alcohol consumption, frequency of consumption and consumption rate. For ex-alcoholic participants, additional questions were asked on how long ago they had quit drinking. ‘Standard drink’ terminology was used in alcohol consumption assessment to gather the comparative assessment information across different alcoholic beverages like beer, spirit, wine/liquor according to WHO, STEP Surveillance. The ‘standard drink’ amount for different alcoholic beverages, as shown in Supplementary 1, was illustrated in the show card and used as a study patient reference to facilitate the assessment process. Data on alcohol consumption did not include drinking a few sips of alcohol for religious or other reasons.

Physical activity levels were assessed using the revised Global Physical Activity Questionnaire (GPAQ) (56). The participants were asked to recall their physical activity before the first symptom on CRC screening or colonoscopy. The participants were asked to report the number of days a week and the total hours spent performing vigorous and/or moderate-intensity activities and sedentary behaviours in three major environments (work, transport and recreation). Expressing the intensity of physical activity and analysing GPAQ-WHO data are commonly performed using METs. MET is the ratio of a person’s working metabolic rate to their resting metabolic rate. One MET is interpreted as the energy cost of sitting quietly, which is similar to a caloric expenditure of 1 kcal/kg/h. Individuals’ caloric burning was four times higher when they performed moderate-intensity activities and eight times higher when they performed vigorous-intensity activities than those associated with sitting quietly. The total hours of physical activity were multiplied with the respective MET values to determine the MET hours per week for each participant. The total physical activity of each participant was then classified as high, moderate, or low according to the corresponding cut-off values (56).

The body mass index (BMI) was used to indicate body fat levels. Study participants with BMI > 30 kg/m^2^ were classified as obese, whereas the remaining participants were classified as normal. The BMI was calculated and classified according to the International Diabetes Federation (IDF) (57).

##### Dietary assessment

The participants’ daily dietary intake for the preceding month was collected by a validated 123-item semiquantitative food frequency questionnaire adapted from the Malaysian Adult Nutrition Survey (58). The researcher conducted a one-to-one, face-to-face interview in which the participants were asked to recall their habitual dietary intake before the first symptom upon CRC screening or colonoscopy. They were asked questions regarding the frequency of food intake and the size of portions consumed according to the food items. Portion size questions were asked using measuring cups and spoons set as support to quantify food and beverage items to estimate the amount consumed, which was later converted to grams using the Food Portion Sizes of Malaysian Foods Album 2002/2003 (58). The frequency of food intake was determined using a 5-point scale from 5 for daily intake, 4 for 2–3 times a week intake, 3 for once-a-week intake, 2 for once-a-month intake and 1 for never have consumed. The conversion of food frequency to food intake was calculated using the following equation, which was previously used in a national surveillance study (58):


$$\begin{array}{c} Amount\;of\;food\;consumed\;per\;day\;\left(\frac{g}{day}\right)\\ =frequency\;of\;intake\;(conversion\;factor)\\ \times \;serving\;size\times total\;number\;of\;servings\\ \times \;weight\;of\;food\;in\;one\;serving \end{array}$$

All nutrient values (grams) calculations of food consumed were conducted using Nutritionist Pro™ Diet Analysis (Axxya Systems, Stafford, TX, USA) software.

### Principle Component Analysis

Food data were analysed using principal component analysis of the factor analysis based on 35 food groups. Varimax rotation was used to improve interpretation and minimise the correlation between the factors. The Keiser-Meyer-Olkin (KMO) measure of sampling adequacy and Bartlett’s test of sphericity were used to assess statistical correlations between variables and sample size adequacy. The dietary patterns were selected using a scree plot (Eigenvalue > 1). The factor loading of each tested food group of the chosen factors was extracted from the ‘rotated component matrix’ table of factor analysis. Foods with absolute factor loading values < 0.15 were excluded for simplicity. Each food group belonged to the particular extracted factor with the highest factor loading. Food groups with positive loadings for each pattern indicated a direct relationship with that pattern, whereas food groups with negative loadings showed an inverse relationship. The total energy intake of each food group was multiplied by the factor loadings. Next, the multiplied values of each food group that belonged to one factor/dietary pattern were grouped by summing the values, known as the factor scores. The final score for each dietary pattern was used as an independent variable for statistical analysis (59).

### Statistical Analysis

Data were analysed using the Statistical Package for Social Sciences for Windows (version 21.0; SPSS, Chicago, IL, USA). The normality of the data was determined using the Kolmogorov-Smirnov test. Descriptive statistics were used to calculate the frequencies, percentages, means, ranges and standard deviations. Normally distributed variables are expressed as means and standard deviations. Absolute numbers and percentages were used to report categorical variables. Comparison of participants’ characteristics and of dietary intake between cases and controls were performed using the Pearson’s chi-square (χ^2^) test or Fisher’s exact test for categorical variables, and the independent *t*-test for continuous variables. Logistic regression analysis was used to assess the OR with a 95% confidence interval (CI) for CRC. All models were adjusted for potential confounding variables. Statistical significance was set at *P* < 0.05.

## Results

### Participant Characteristics

**Figure 1:**
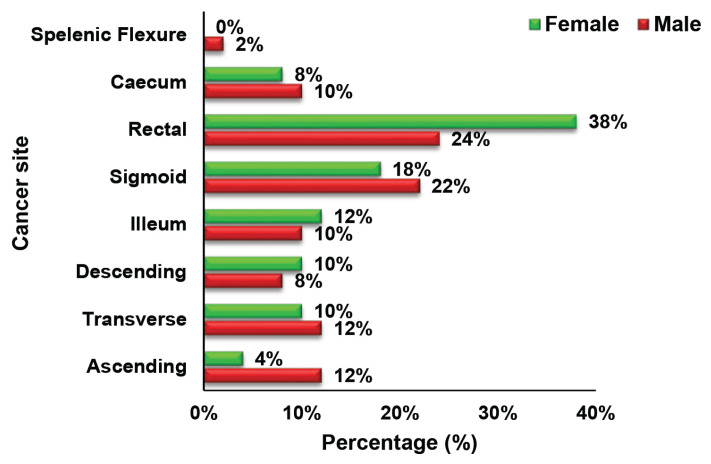
Distribution of cancer sites by gender among CRC patients

**Figure 2:**
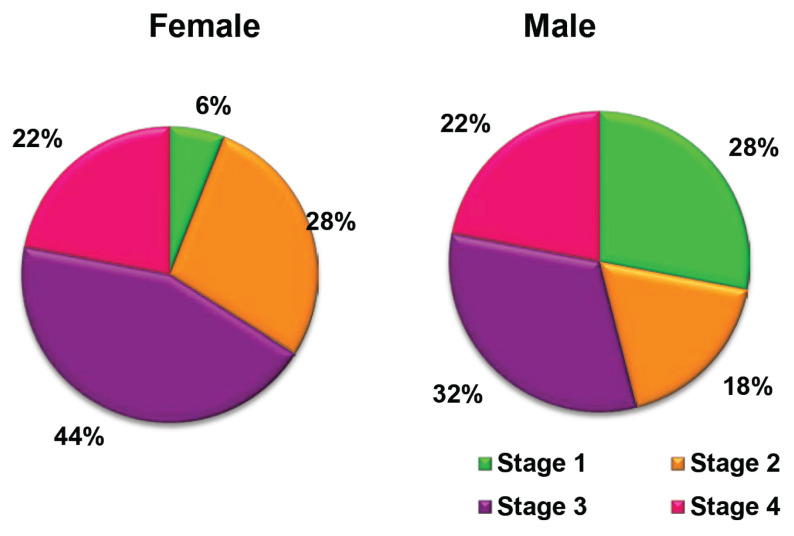
Distribution of cancer stage by gender among CRC patients

The socioeconomic characteristics, educational background, and family and medical histories of the 100 cases and 100 controls are shown in Table 1. Using a frequency-matched design, the age, ethnicity and sex distributions of the cases and controls were similar for both groups. Most participants in the case group were unemployed compared to those in the control group. The groups did not differ significantly in terms of marital status, family history of chronic disease, or family history of cancer. Conversely, the chi-square test revealed a significant difference in the educational background (χ^2^ = 9.995, *P* = 0.041) and cancer indicators (χ^2^ = 19.752, *P* < 0.001) between the groups. Most case participants (44%) had lower educational levels than those of the control group (28%). Participants who portrayed a few indications underwent a colonoscopy to confirm the diagnosis. The CRC symptoms commonly found among the case participants included alterations in bowel habits, pre-rectal bleeding, anaemia and melena. However, controls who underwent colonoscopy had diarrhoea as the most common symptom. Meanwhile, constipation was a common symptom for both case and control participants. The most prevalent sites of cancer growth in both male and female CRC patients were the rectum and sigmoid colon. This was followed by the ascending and transverse colons as the third most frequent cancer site for men and the ileum for women. No significant association was found between the cancer site and CRC patients’ sex (χ^2^ = 0.228, *P* = 0.637) (Figure 1). Nevertheless, the data showed that most of the CRC patients were diagnosed with Stage III cancer (men: 32%; women: 44%), followed by Stage I cancer in men (28%), and Stage II cancer in women (28%) (χ^2^ = 9.152, *P* = 0.027), as shown in Figure 2.

### Dietary Patterns

The KMO value was 0.601 and the significance value for Bartlett’s test of sphericity was 0.001 (*P* < 0.05), which indicates that factor analysis is useful for this data. Thirteen components with an Eigenvalue greater than 1.0 were found. However, only three dietary patterns were identified through a scree plot. The food groups of each dietary pattern and their factor loadings are presented in Table 2. After rotation, the three identified dietary patterns explained 27.48% of the total variance in food consumption, with the first pattern accounting for 12.42%, the second for 8.86% and the third for 6.20%. The first pattern was named the ‘vegetable’ dietary pattern, with high loadings of leafy vegetables, cucurbit, bean vegetables, root vegetables, mushrooms and fruits. The second pattern, which had high loadings of salty food, poultry, shellfish, crustaceans, pork, dairy product, processed meat and processed seafood, was labelled as the ‘meat, seafood and processed food’ dietary pattern. The last pattern, loaded highly in nuts, tempeh, legumes, soy and cereals, was named the ‘grains and legumes’ dietary pattern (Table 2).

We identified a significant association between the ‘vegetable’ dietary pattern and study groups (χ^2^ = 17.952, *P* = 0.001). With this, 18% of the case participants and 33% of the control participants consumed a low ‘vegetable’ dietary pattern (first quartile, < 51.59 calories/day). However, 37% of the case participants and 13% of the control participants consumed a high ‘vegetable’ diet (fourth quartile, > 131.00 calories/day). Dietary patterns of ‘meat, seafood and processed food’ and of ‘grains and legumes’ were not significantly associated with the study groups (Table 3). Low ‘vegetable’ diet intake (first quartile) was independently and significantly associated with a 64% decreased risk of CRC (OR = 0.36; 95% CI: 0.15, 0.84). Meanwhile, high ‘vegetable’ intake (third quartile) was independently and significantly associated with an 81% decreased risk of CRC (OR = 0.19; 95% CI: 0.08, 0.46). Conversely, a high intake of ‘meat, seafood and processed food’ (fourth quartile) independently and non-significantly increased CRC risk (OR = 1.17; 95% CI: 0.52, 2.66), while a high intake of ‘grains and legumes’ dietary pattern (third quartile) independently and non-significantly decreased 51% CRC risk (OR = 0.49; 95% CI: 0.15, 1.62) (Table 4).

### Lifestyle Factors

Table 3 presents the mean physical activity (PA) practices at home, workplace and outdoors. The *t*-test shows a significant difference in the mean metabolic equivalent of task (MET) minutes spent per day for recreation-related PA, in which case participants have lower average mean MET minutes than control participants. The chi-square test showed significant differences between the study groups for both recreational PA (χ^2^ = 5.851, *P* = 0.016) and vigorous PA (χ^2^ = 5.433, *P* = 0.020), whereas work- and transport-related PA showed no significant association. There are significant risk patterns for both recreational-related PA (OR = 2.04; 95% CI: 1.14, 3.64) and vigorous PA (OR = 2.06; 95% CI: 1.13, 3.74) independently reduce the risk of CRC by more than two-fold meanwhile work and transport-related PA showed statistically non-significant association with risk of CRC (Table 4).

No notable risk patterns were found for current smoking status, past smoking status, or duration of smoking cessation on the contribution to CRC risk, as shown in Table 4. When the number of cigarettes smoked daily is separated into a few levels, only ≥ 16 cigarettes smoked per day showed an independent and significantly increased risk of CRC (OR = 2.58; 95% CI: 1.95, 6.75). However, the analysis showed an increased risk pattern with an increase in the number of smoked cigarettes. However, for the number of cigarettes smoked per day previously, only 6–15 cigarettes smoked daily showed an independent and significantly increased risk of CRC (OR = 1.43; 95% CI: 1.15, 4.56) (Table 4).

No significant risk pattern was found for CRC risk based on current alcohol consumption status, past alcohol consumption status, frequency of current alcohol consumption or frequency of past alcohol consumption, as shown in Table 4. Duration of quitting alcohol consumption showed a significantly increased risk pattern where participants who quit alcohol consumption for less than 1 year had significantly increased risk of CRC for almost three folds (OR = 2.52; 95% CI: 2.30, 10.57) when compared to those participants who quit alcohol consumption for more than 4 years as the former had 72% of increased risk of CRC (OR = 1.72; 95% CI: 0.39, 7.62).

## Discussion

In the present study, CRC was detected in 54% of male and 66% of female patients at late stages (stages 3 and 4), and most CRC patients had pre-rectal bleeding and constipation symptoms. This result aligns with current Malaysian CRC studies (60–62). The results revealed that most patients seek medical treatment at an advanced stage of CRC, when they have already developed pre-rectal bleeding symptoms. This could have been due to a lack of cancer-related knowledge, which is an upcoming chronic disease among Malaysians. Efficient campaigns should be undertaken to improve knowledge and health-seeking attitudes to improve disease prognosis (63).

This study demonstrated that high fruit and vegetable consumption has a protective effect against CRC. The rationale may be that they are rich in natural components such as vitamins, polyphenols, PUFA and dietary fibre. Antioxidants present in this food group may reduce CRC risk by quenching free radicals and reducing oxidative DNA damage (64). Fibres also contribute to a healthier gastrointestinal system by diluting faecal content, decreasing transit time, and increasing stool weight (65). Animal studies have examined these mechanisms. The diet can reshape the community structure of the gut microbiota and alter its function by influencing metabolite synthesis. Butyrate, a four-carbon short-chain fatty acid, is produced in the lower intestinal tract by microbial fermentation of dietary fibre and may protect colonic epithelial cells from tumorigenesis through anti-inflammatory and antineoplastic properties mediated by cell metabolism, microbiota homeostasis, and antiproliferative, immunomodulatory and genetic/epigenetic regulation (66). Despite the multiple biological and chemical mechanisms that indicate the protective effect of fruit and vegetable consumption against CRC, observational studies are yet to substantiate this association (67, 68). Conversely, an American study showed that high vegetable and fruit intake reduced the risk of CRC by 19% among male participants (69). This finding was supported by a North Carolina case-control study that negatively correlated a high vegetable intake with the risk of CRC (70). Another study in the United Kingdom showed a 43% decreased risk of CRC in individuals with high fruit consumption (71). A meta-analysis correlated a lower risk of CRC with dietary patterns with high fruit and vegetable intakes (72).

The dietary patterns of ‘meat, seafood and processed food’, and ‘grains and legumes’ were not associated with CRC risk. These results conform with those of a prospective study conducted in Europe (73) and a cohort study in the United States (74). However, processed meat consumption had showed a direct correlation with increased risk of colon cancer by 33% (74). A meta-analysis of 22 cohort and case-control studies reported that consuming more than 50 g of red meat daily had a positive association with increased risk of colon cancer by 21% but no association was observed with rectal cancer (75). A Malaysian qualitative study of other dietary patterns stated that Western diet consumption was associated with increased risk of CRC due to high preservative chemical usage, in contrast with a traditional Malaysian diet, which was not associated with CRC risk (76). Another study in Tehran reported that participants with a highly healthy dietary pattern had a reduced risk of CRC by 77%, while a Western dietary pattern almost tripled the risk of CRC (59).

Both recreational PA and vigorous PA showed a strong direct association with an increased risk of CRC occurrence. Conversely, work-related PA and transport-related PA showed no significant contribution. However, these findings remain controversial in the current studies. A systematic meta-analysis by Mahmood et al. (77) suggested that higher PA in terms of recreational activity was associated with a reduced risk of CRC. Another retrospective case-control study supported these outcomes by comparing the highest and lowest PA categories among Vietnamese adults (7). Healthy physical movements and regular physical exercise effectively reduce obesity and maintain a healthy lifestyle, which has been consistently correlated with CRC risk (7). Although this study found a strong positive association between CRC risk and recreational and leisure-related PA, the case group had very low recreational and leisure-related PA. Healthy physical movement and regular physical exercise effectively reduce obesity and maintain a healthy life (78), as lifestyle has consistently been correlated with CRC risk (5).

Current smokers who smoked more than 16 cigarettes daily showed a strong direct association with CRC risk. In line with this finding, a meta-analysis of 24 prospective studies reported that smokers who consume 10 additional cigarettes daily may increase their CRC risk by 7.8%. The same study mentioned that the CRC risk increased by 4.4% for the additional smoking of 10 packs yearly (79). In another study, smokers who smoked 20 cigarettes per day showed a positive association with increased risk of CRC by 17.5% and those who smoked 40 cigarettes per day had a 38% increased risk of CRC (80). Several potential mechanisms could explain the role of cigarette smoking in CRC development, which have been described in many scientific reports (81–85). Cigarettes contain more than 7,000 toxic chemicals, including carcinogens such as polycyclic aromatic hydrocarbons, nitrosamines, heterocyclic amines, aromatic amines and benzene, which may reach the colorectal mucosa via direct ingestion or the bloodstream and can cause CRC (81). In vivo and human studies have found a significant association between polycyclic aromatic hydrocarbons and heterocyclic amines and the risk of CRC (86, 87).

Quitting alcohol consumption for less than 1 year had a significant direct association with the risk of CRC. The prolonged effects of alcohol consumption and the duration required to reduce the adverse effects, even after quitting, remain unclear. Some studies have shown that an average daily alcohol consumption of 30 g/day–45 g/day is directly associated with the risk (31, 88–90). Although the negative effects of ethanol have been widely established, the biological mechanisms by which alcohol causes CRC remain unclear. Alcohol metabolism involves the conversion of ethanol into its metabolites, which can potentially cause colon cancer. The colon microbiota, another recently established mediating factor in colon carcinogenesis, can influence the production of ethanol metabolites. Several cancer-promoting pathways, including DNA adduct formation, oxidative stress, lipid peroxidation, epigenetic alterations, epithelial barrier dysfunction and immunological modulatory effects, are activated by the production of acetaldehyde and other alcohol metabolites. In addition to the carcinogenic metabolites with negative effects, alcohol consumers are predisposed to a poor diet deficient in folate and fibre, as well as circadian disruption, which may increase the likelihood of alcohol causing colon carcinogenesis (42).

The strengths of this study include the use of validated questionnaires and that the analyses controlled for all confounding factors identified in the literature. Moreover, this was a case-control study in Malaysia, which has a multi-ethnic and multi-food culture. Studies in multiracial and multicultural countries can provide unique opportunities to test the association between dietary patterns and cancer. Malaysia likely has a unique set of dietary patterns and genetic variances in susceptibility, which can be used to examine the association between diet and disease (84).

Nevertheless, this study had several limitations. For example, information bias might have occurred. Some participants might have changed their diet or lifestyle after the onset of their first CRC symptoms. This situation might not have been reported to the researcher and might have influenced the accuracy of the data. However, the participants had never received dietary counselling from a medical officer or dietician. Thus, we assumed that the participants provided accurate information that was not influenced by any external knowledge. Some selection bias might have occurred. The cases and controls were matched for age, sex and ethnicity; however, not all controls were from the same hospitals where the cases were admitted. Differences in environmental exposure between the cases and controls might have altered the study outcomes.

## Conclusion

The current study developed knowledge of the pattern of food consumption, as indicated by the dietary pattern of fruits and vegetables, which may be used as a dietary recommendation to reduce the risk of CRC. The significant relationship between CRC risk and lifestyle factors reiterates the previously published information. Lifestyle changes in dietary intake, quitting cigarette smoking, and reducing alcohol intake could serve as important ways to prevent CRC and these modifiable risk factors could be treated with a healthy lifestyle and dietary intake.

## Supplementary Data

**Supplementary 1:** Quantities of one standard drink of alcoholic beverages

Types of alcoholic beverages	Volume	Content of ethanol	Ethanol	Standard drink
Shandy	1 can-330 mL	0.5%–1.0%	15g	0.5
Beer	1 bottle/can-330 mL	4.0%–5.0%	15g	1.5
Wine/liquor	1 glass-100 mL	12.5%	12.5g	1.3
Spirit/Vodka	1 glass-50 mL	40%	20g	2.0

## Figures and Tables

**Table 1:** Characteristics of the subjects

Variables	Case (*N* = 100) *N* (%)	Control (*N* = 100) *N* (%)	χ^2^	*P*-value
Mean age ± SD (years old)	60.21 ± 11.04	58.69 ± 10.95		
Range (years)	37–79	35–77	–	–
Ethnicity				
Malay	41 (41)	42 (42)	–	–
Chinese	42 (42)	42 (42)		
Indian	17 (17)	16 (16)		
Gender				
Male	50 (50)	50 (50)	–	–
Female	50 (50)	50 (50)		
Educational background				
Primary school	44 (44)	28 (28)	9.995	* **0.041**
Secondary school	30 (30)	44 (44)		
Certificates/STPM	6 (6)	9 (9)		
Tertiary	8 (8)	13 (13)		
No schooling	12 (12)	6 (6)		
Current occupation				
Employed	43 (43)	57 (57)	7.429	0.115
Un-employed	41 (41)	27 (27)		
Retired/ pension	16 (16)	16 (16)		
Marital status				
Single	5 (5)	8 (8)	2.382	0.304
Married	85 (85)	87 (87)		
Widow/widower	10 (10)	5 (5)		
Body mass index (BMI)				
Underweight	7 (7)	9 (9)	0.113	0.765
Normal	51 (51)	49 (49)		
Overweight	29 (29)	34 (34)		
Obese class I	11 (11)	7 (7)		
Obese class II	1 (1)	1 (1)		
Obese class III	1 (1)	0		
Family history				
Chronic diseases				
No	33 (33)	42 (42)	1.728	0.189
Yes	67 (67)	58 (58)		
Cancer				
No	76 (76)	80 (80)	0.466	0.495
Yes	24 (24)	20 (20)		
Cancer indication: Signs and symptoms				
Alteration in bowel habits	17 (17)	8 (8)	19.752	** **0.001**
Pre-rectal bleeding	33 (33)	26 (26)		
Constipation	21 (21)	20 (20)		
Diarrhoea	11 (11)	36 (36)		
Melena	8 (8)	5 (5)		
Anaemia	10 (10)	5 (5)		

Notes: χ^2^ = chi-square; *P*-value:

* *P* < 0.05 = significant value,

** *P* < 0.001 = statistically significant;

SD = standard deviation

**Table 2:** Factor loading matrix of food groups for vegetables, meat, seafood and processed food, and grains and legumes

Food groups	Dietary patterns			Communality

	Vegetables	Meat, seafood and processed food	Grains and legumes	
Leafy vegetable	0.874			0.742
Cucurbit	0.830		0.201	0.593
Beans vegetable	0.850			0.832
Root vegetable	0.774			0.810
Mushroom	0.646			0.794
Fruits	0.256			0.579
Shellfish		0.844		0.773
Crustaceans		0.811		0.760
Processed meat		0.674		0.643
Salted food	0.154	0.645		0.712
Processed seafood		0.361		0.618
Dairy product		0.265		0.535
Pork	0.201	0.204		0.669
Poultry		0.191	–0.154	0.635
Tempeh			0.877	0.792
Nuts			0.856	0.819
Legume	0.312		0.636	0.601
Soy			0.311	0.582
Cereal			0.183	0.702
Eigenvalue	4.346	3.103	2.170	
% of variance explained	12.416	8.864	6.201	
Total variance explained	27.481			

Note: Absolute factor loading values < 0.15 for all three dietary pattern were excluded for simplicity

**Table 3:** Dietary patterns and lifestyle factors of case and control subjects

Characteristics		Case (*N* = 100) *N* (%)	Control (*N* = 100) N (%)	*t*	χ^2^	*P*-value
Vegetable dietary pattern	First (lowest) (values: < 51.59)	18 (18)	33 (33)		17.952	* **0.001**
	Second (values: 51.60–83.29)	20 (20)	30 (30)			
	Third (values: 83.30–130.99)	25 (25)	24 (24)			
	Fourth (highest) (values: > 131.00)	37 (37)	13 (13)			
Meat, seafood and processed food dietary pattern	First (lowest) (values: < 38.49)	23 (23)	27 (27)		0.480	0.923
	Second (values: 38.50–74.59)	26 (26)	24 (24)			
	Third (values: 74.60–148.79)	25 (25)	25 (25)			
	Fourth (highest) (values: > 148.80)	26 (26)	24 (24)			
Grains and legumes dietary pattern	First (lowest) (values: < 25.19)	20 (20)	30 (30)		3.360	0.339
	Second (values: 25.20–60.99)	25 (25)	25 (25)			
	Third (values: 61.00–172.59)	29 (29)	21 (21)			
	Fourth (highest) (values: > 172.6)	26 (26)	24 (24)			
Total PA (METS-minutes/week)	Mean ± SD	1212.75 ± 3473.70	957.29 ± 1169.01	−0.697		0.487
Average time per day of PA (METS-minutes/day)	Mean ± SD	132.31 ± 231.28	138.19 ± 170.36	0.205		0.838
Total PA	High PA [*N* (%)]	35 (35)	27 (27)		2.381	0.304
	Moderate PA [*N* (%)]	31 (31)	29 (29)			
	Low PA [*N* (%)]	34 (34)	44 (44)			
Work related PA	Mean ± SD	89.06 ± 205.22	70.79 ± 133.32	−0.747		0.456
	Yes [*N* (%)]	51 (51)	55 (55)		0.321	0.571
	No [*N* (%)]	49 (49)	45 (45)			
Transport related PA	Mean ± SD	31.56 ± 50.38	47.13 ± 82.85	1.605		0.110
	Yes [*N* (%)]	86 (86)	81 (81)		0.907	0.341
	No [*N* (%)]	14 (14)	19 (19)			
Recreational related PA	Mean ± SD	11.68 ± 24.60	20.28 ± 39.31	1.855		*0.045
	Yes [*N* (%)]	36 (36)	53 (53)		5.851	* **0.016**
	No [*N* (%)]	64 (64)	47 (47)			
Vigorous PA	Mean ± SD	404.40 ± 201.23	387.30 ± 172.33	−0.645		0.519
	Yes [*N* (%)]	30 (30)	46 (46)		5.433	* **0.020**
	No [*N* (%)]	70 (70)	54 (54)			
Current smoking status	No [*N* (%)]	77 (77)	82 (82)		0.767	0.381
	Yes [*N* (%)]	23 (23)	18 (18)			
Number of cigarettes smoked per day currently	Mean ± SD	5.32 ± 13.80	2.20 ± 5.74	2.088		*0.039
	Non-smoker [*N* (%)]	77 (77)	82 (82)		0.169^a^	0.128
	< 6 [*N* (%)]	2 (2)	6 (6)			
	6–15 [*N* (%)]	5 (5)	5 (5)			
	≥ 16 [*N* (%)]	16 (16)	7 (7)			
Past smoking status	No [*N* (%)]	73 (73)	77 (77)		0.427	0.514
	Yes [*N* (%)]	27 (27)	23 (23)			
Number of cigarettes smoked per day previously	Mean ± SD	5.23 ± 11.85	3.94 ± 10.55	0.813		0.417
	Non-smoker [*N* (%)]	73 (73)	77 (77)		0.083	0.707
	< 6 [*N* (%)]	5 (5)	7 (7)			
	6–15 [*N* (%)]	8 (8)	6 (6)			
	≥ 16 [*N* (%)]	14 (14)	10 (10)			
Duration of quitting smoking (years)	Non-smoker [N (%)]	73 (73)	77 (77)		0.105^a^	0.534
	< 1 [*N* (%)]	4 (4)	1 (1)			
	1–3 [*N* (%)]	9 (9)	7 (7)			
	≥ 4 [*N* (%)]	14 (14)	15 (15)			
Current alcohol consumption status	No [*N* (%)]	80 (80)	79 (79)		0.031	0.861
	Yes [*N* (%)]	20 (20)	21 (21)			
Number of standard drink consumed during one occasion currently	Mean ± SD	0.39 ± 1.20	0.56 ± 1.67	0.827		0.409
Frequency of current alcohol consumption (at least one alcohol drink)	Non-drinker	81 (81)	79 (79)		0.130^a^	0.334
	1–3 days/month	12 (12)	13 (13)			
	1–5 days/week	7 (7)	5 (5)			
	6–7 days/week	1 (1)	3 (3)			
Past alcohol consumption status	No [*N* (%)]	72 (72)	74 (74)		0.101	0.750
	Yes [*N* (%)]	28 (28)	26 (26)			
Number of standard drink consumed during one occasion previously	Mean ± SD	1.52 ± 2.87	1.04 ± 2.68		−1.223	0.223
Frequency of past alcohol consumption (at least one alcohol drink)	Non-drinker	71 (71)	74 (74)		0.124	* **0.043**
	1–3 days/month	12 (12)	12 (12)			
	1–5 days/week	10 (10)	8 (8)			
	6–7days/week	6 (6)	6 (6)			
Duration of quitting alcohol consumption (years)	< 1	6 (6)	8 (8)		0.175	* **0.021**
	1–3	13 (13)	11 (11)			
	≥ 4	9 (9)	7 (7)			

Notes: *t*-value = independent sample *t*-test; χ^2^ = chi-square; *P*-value = significant value;

* *P* < 0.05 = statistically significant;

a Fisher exact tests;

* PA = physical activity

**Table 4:** Dietary patterns and lifestyle factors of case and control subjects and its association to CRC risk

Characteristics		OR (95% Cl)	Adjusted OR (95% Cl)
Vegetable dietary pattern	Fourth (highest) (values: > 131.00)	1	1
	Third (values: 83.30–130.99)	0.19 (0.08, 0.45)	*0.19 (0.08, 0.46)
	Second (values: 51.60–83.29)	0.23 (0.10, 0.55)	*0.24 (0.10, 0.55)
	First (lowest) (values: < 51.59)	0.37 (0.16, 0.85)	*0.36 (0.15, 0.84)
Meat, seafood and processed food dietary pattern	First (lowest) (values: < 38.49)	1	1
	Second (values: 38.50–74.59)	1.27 (0.58, 2.79)	1.26 (0.55, 2.87)
	Third (values: 74.60–148.79)	1.17 (0.54, 2.57)	1.11 (0.49, 2.48)
	Fourth (highest) (values: > 148.80)	1.27 (0.58, 2.79)	1.17 (0.52, 2.66)
Grains and legumes dietary pattern	Fourth (highest) (values: > 172.6)	1	1
	Third (values: 61.00–172.59)	0.62 (0.28, 1.36)	0.49 (0.15, 1.62)
	Second (values: 25.20–60.99)	0.92 (0.42, 2.02)	0.61 (0.27, 1.37)
	First (lowest) (values: < 25.19)	1.28 (0.58, 2.81)	0.95 (0.43, 2.11)
Average time per day of PA (METS-min/day)			
Total PA	High PA		
	Moderate PA	1.38 (0.70, 2.72)	1.42 (0.71, 2.85)
	Low PA	1.68 (0.86, 3.29)	1.67 (0.85, 3.32)
Work-related PA	Yes		
	No	1.17 (0.67, 2.05)	1.15 (0.65, 2.02)
Transport-related PA	Yes		
	No	1.69 (0.33, 1.48)	0.68 (0.31, 1.45)
Recreational-related PA	Yes		
	No	2.01 (1.14, 3.53)	*2.04 (1.14, 3.64)
Vigorous PA	Yes		
	No	1.99 (1.11, 3.55)	*2.06 (1.13, 3.74)
Current smoking status	No		
	Yes	1.36 (0.68, 2.72)	1.42 (0.67, 3.01)
Number of cigarettes smoked per day currently	Non-smoker		
	< 6	0.36 (0.07, 1.81)	0.37 (0.07, 1.98)
	6–15	1.07 (0.30, 3.82)	1.09 (0.29, 4.05)
	≥ 16	2.43 (0.95, 6.24)	*2.58 (1.95, 6.75)
Past smoking status	No		
	Yes	1.24 (0.65, 2.35)	1.26 (0.62, 2.58)
Number of cigarettes smoked per day previously	Non-smoker		
	< 6	0.63 (0.11, 3.67)	0.77 (0.22, 2.68)
	6–15	1.07 (0.20, 5.67)	*1.43 (1.15, 4.56)
	≥ 16	1.19 (0.30, 4.80)	1.51 (0.59, 3.85)
Duration of quitting smoking (years)	< 1	4.22 (0.46, 38.64)	4.32 (0.45, 41.53)
	1–3	1.36 (0.48, 3.83)	1.49 (0.50, 4.47)
	≥ 4	0.98 (0.44, 2.18)	0.98 (0.42, 2.32)
Current alcohol consumption status	No		
	Yes	1.94 (0.47, 1.87)	1.83 (0.30, 2.27)
Frequency of current alcohol consumption (at least one alcohol drink)	Non-drinker		
	1–3 days/month	0.90 (0.39, 2.09)	0.93 (0.38, 2.31)
	1–5 days/week	1.14 (0.23, 5.67)	1.38 (0.40, 4.72)
	6–7days/week	1.37 (0.42, 4.48)	1.65 (0.36, 6.12)
Past alcohol consumption status	No		
	Yes	1.11 (0.59, 2.07)	1.15 (0.56, 2.33)
Frequency of past alcohol consumption (at least one alcohol drink)	Non-drinker		
	1–3 days/month	1.04 (0.32, 3.38)	1.20 (0.49, 2.97)
	1–5 days/week	1.13 (0.48, 2.64)	1.36 (0.48, 3.89)
	6–7days/week	1.30 (0.49, 3.49)	1.39 (0.29, 3.60)
Duration of quitting alcohol consumption (years)	Non-drinker < 1	2.09 (0.19, 13.44)	*2.52 (2.30, 10.57)
	1–3	1.74 (0.40, 7.50)	1.50 (0.24, 9.55)
	≥ 4	1.57 (0.26, 9.60)	1.72 (0.39, 7.62)

Notes: PA = physical activity; METS = metabolic equivalents; OR = estimates of crude odds ratio from binary logistic regression equations; Adjusted OR = estimates of crude odds ratio from binary logistic regression equations including terms of age, sex, ethnic, education background; BMI = body mass index, smoking status and alcohol consumptions status; CI = confidence interval
